# *Histophilus somni* Stimulates Expression of Antiviral Proteins and Inhibits BRSV Replication in Bovine Respiratory Epithelial Cells

**DOI:** 10.1371/journal.pone.0148551

**Published:** 2016-02-09

**Authors:** C. Lin, J. T. Agnes, N. Behrens, M. Shao, Y. Tagawa, L. J. Gershwin, L. B. Corbeil

**Affiliations:** 1 Department of Pathology, University of California San Diego, San Diego, CA, United States of America; 2 Department of Pathology, Microbiology, and Immunology, School of Veterinary Medicine, University of California Davis, Davis, CA, United States of America; 3 Bacterial and Parasitic Diseases Research Division, National Institute of Animal Health, NARO, Tsukuba, Ibaraki, Japan; 4 Department of Population Health and Reproduction, School of Veterinary Medicine, University of California Davis, Davis, CA, United States of America; Cornell University, UNITED STATES

## Abstract

Our previous studies showed that bovine respiratory syncytial virus (BRSV) followed by *Histophilus somni* causes more severe bovine respiratory disease and a more permeable alveolar barrier *in vitro* than either agent alone. However, microarray analysis revealed the treatment of bovine alveolar type 2 (BAT2) epithelial cells with *H*. *somni* concentrated culture supernatant (CCS) stimulated up-regulation of four antiviral protein genes as compared with BRSV infection or dual treatment. This suggested that inhibition of viral infection, rather than synergy, may occur if the bacterial infection occurred before the viral infection. Viperin (or radical S-adenosyl methionine domain containing 2—RSAD2) and ISG15 (IFN-stimulated gene 15—ubiquitin-like modifier) were most up-regulated. CCS dose and time course for up-regulation of viperin protein levels were determined in treated bovine turbinate (BT) upper respiratory cells and BAT2 lower respiratory cells by Western blotting. Treatment of BAT2 cells with *H*. *somni* culture supernatant before BRSV infection dramatically reduced viral replication as determined by qRT PCR, supporting the hypothesis that the bacterial infection may inhibit viral infection. Studies of the role of the two known *H*. *somni* cytotoxins showed that viperin protein expression was induced by endotoxin (lipooligosaccharide) but not by IbpA, which mediates alveolar permeability and *H*. *somni* invasion. A naturally occurring IbpA negative asymptomatic carrier strain of *H*. *somni* (129Pt) does not cause BAT2 cell retraction or permeability of alveolar cell monolayers, so lacks virulence *in vitro*. To investigate initial steps of pathogenesis, we showed that strain 129Pt attached to BT cells and induced a strong viperin response *in vitro*. Thus colonization of the bovine upper respiratory tract with an asymptomatic carrier strain lacking virulence may decrease viral infection and the subsequent enhancement of bacterial respiratory infection *in vivo*.

## Introduction

Human or bovine respiratory disease often has a multifactorial etiology. Interactions between viruses and bacteria are known to cause more severe disease than either one alone even though both may colonize the upper respiratory tract without causing disease [1–3]. Several studies have revealed mechanisms of viral predisposition to bacterial pneumonia [1, 3]. We have investigated viral bacterial interactions in bovine respiratory disease (BRD) which is caused by a variety of pathogens including the following bacteria: *Mannheimia haemolytica*, *Pasteurella multocida*, *Mycoplasma bovis* and *Histophilus somni* and viruses: bovine respiratory syncytial virus (BRSV), bovine viral diarrhea virus (BVDV), bovine parainfluenza 3 (BPIV-3) and bovine herpes virus 1 (BHV-1). Our team has reproduced experimental pneumonia in calves with either *H*. *somni* [4–6] or BRSV alone [7–10]. Studies of interactions of BRSV and *H*. *somni* showed that aerosol infection of calves with BRSV 6 days before intrabronchial inoculation of *H*. *somni*, resulted in more severe pneumonia of longer duration than either pathogen alone [11]. This was associated with higher increases in specific IgE anti-*H*. *somni* levels in the dual infection than in either single infection [11]. Since BRSV and *H*. *somni* infect both upper respiratory and lower respiratory tract cells *in vivo* and *in vitro*, we have used both bovine turbinate cells (BT) and bovine alveolar type 2 (BAT2) epithelial cells for further investigation of pathogenic mechanisms. To study bacterial viral synergy, BAT2 cells were infected with BRSV 60 h before treatment with *H*. *somni* concentrated culture supernatant (CCS) for 4 h. This resulted in increased retraction of the BAT2 cells and microarray analysis showed increased BAT2 cell expression of matrix metalloproteinase (MMP)1 and MMP3 over either treatment alone [12]. The dual treatment of BAT2 cells increased passage of *H*. *somni* across the alveolar cell monolayer and increased digestion of collagen IV, a major component of the alveolar basement membrane. Thus, dual infection facilitated invasion by the bacteria [12]. We found that the IbpA was the major factor in CCS which caused retraction of BAT2 cells [13]. IbpA consists of a surface fibrillar network that is released into culture supernatant from all strains isolated from disease and most strains from asymptomatic carriers [14, 15]. However, the *ibpA* gene was missing in four serum sensitive strains from asymptomatic preputial carriers (1P, 129Pt, 130Pf and 133P) [16]. The complete genome sequence of one of these IbpA negative asymptomatic carrier stains (129Pt) has been reported [17]. IbpA from disease isolates of *H*. *somni* has two direct repeats (DR1 and DR2), each with a cytotoxic fic motif which adenylylates Rho GTPases interfering with the cytoskeleton [18]. The motivation for the current study came from the observation that treatment of BAT2 cells with *H*. *somni* CCS as described above increased mRNA expression of four antiviral proteins over that of either BRSV or dual treated cells. Viperin (virus-inhibitory protein, endoplasmic reticulum associated, IFN-inducible) or RSAD2 (radical S-adenosyl methionine domain containing 2) and ISG15 (IFN-stimulated gene 15—ubiquitin-like modifier) were the most up-regulated antiviral genes. Therefore we hypothesized that release of *H*. *somni* factors on the surface of respiratory epithelial cells before viral infection may inhibit subsequent viral infection, the opposite of synergy. To test this hypothesis, we investigated up-regulation of viperin protein in BAT2 cells and BT cells as well as the role of *H*. *somni* toxins on increasing expression of antiviral proteins. To address antiviral function, we examined the effect of CCS treatment of BAT2 cells on BRSV replication and mechanisms of up-regulation of antiviral genes. Lastly, adherence to BT cells was investigated because *H*. *somni* does colonize the bovine upper respiratory tract *in vivo* [19], does form biofilms *in vitro* as well as *in vivo* and adherence is a step in biofilm formation [20, 21]. Sustained adherence to the epithelial surface would allow continuous release of *H*. *somni* secreted products which stimulate increased expression of antiviral proteins.

## Materials and Methods

### Bacteria

Pathogenic *H*. *somni* strain 2336 and asymptomatic carrier *H*. *somni* strain 129Pt, which have been previously described [4, 16, 17], were grown on Difco BHI agar (BD Diagnostics, Sparks, MD) plates with 5% bovine blood in Alsever's solution (Lampire Biological Laboratories, Pipersville, PA) in candle jars at 37°C. Strain 2336.A1, with the *ibpA* gene deleted [22], was grown on BHI blood agar supplemented with Kanamycin (145 μg/ml). Growth from 18 h plates was suspended in BHI broth containing 0.1% Tris base and 0.01% thiamine monophosphate (BHITT). For studies of interaction of live bacteria with BT or BAT2 cells, the cfus of bacteria were estimated spectrophotometrically, since 75% light Transmission at 610 nm equals approximately 2 x10^8^ cfu/ml. Concentrated culture supernatant (CCS) was prepared by incubating *H*. *somni* for 7 h in BHITT, with an estimated starting concentration of 5 x 10^7^ cfu/ml. After 7 h of incubation, (approximately late log phase) the culture supernatant was concentrated 40 times with Amicon Ultra-15 Centrifugal Filter Units (EMD Millipore, Billerica, MA) and filtered through 0.22 um sterile syringe filters (EMD Millipore, Billerica, MA) as previously described [12].

### Bovine Respiratory Syncytial Virus preparation

A virulent clinical isolate of BRSV (strain CA-1) was propagated in primary bovine turbinate (BT) cells as previously described (12). A 1ml aliquot was used for measuring the plaque forming units (PFU) /ml and the rest stored at -80°C in aliquots of 1ml in BT cell media containing 10% horse serum and transferred to liquid nitrogen after 2–4 hours.

### BAT2 and BT cell culture

Primary bovine alveolar type 2 (BAT2) cells were isolated from newborn calf lung and kindly provided by Riccardo Rosenbusch (Iowa State University) as previously described [13] and approved by the Institutional Animal Care and Use Committee at Iowa State University. They were grown in DMEM/Defined Keratinocyte-SFM (1:1, Life Technologies, Grand Island, NY) supplemented with 2% gamma-irradiated FBS, 5 mM L-glutamine (Life Technologies, Grand Island, NY), 0.02% lactalbumin dehydrogenase (Life Technologies, Grand Island, NY) for up to 13 passages. Bovine turbinate (BT) cells (ATCC, Manassas, VA) passages 8 and 9 from a bovine turbinate cell line, were grown in DMEM containing 10% horse serum (Hyclone, ThermoFisher Scientific, Waltham, MA). Penicillin (100U/mL) and streptomycin (100 ug/mL) (Life Technologies) were added to media of both cell types, unless cells were to be treated with live *H*. *somni*. Both cell types were grown at 37°C with 5% CO_2_ in flasks or plates coated with 0.1% porcine gelatin (Sigma, St. Louis, MO). When appropriate, the Trypan Blue exclusion assay was used to measure cell viability. The cell suspension was mixed with an equal volume of 0.4% trypan blue solution (ThermoFisher Scientific, Waltham, MA). Viable cells, which did not take up the dye, were counted using a hemocytometer under 20x magnification. For assays other than the microarray studies, 12 well plates (Costar, Corning, Tewksbury, MA) were seeded at 3 x10^4^ cells per well and grown to confluence before treatment with *H*. *somni* or its secreted products as described in the results section. At the indicated time points, the medium was removed and cells were immediately lysed with 100ul/well protein lysis buffer (150mM NaCl, 1% Triton X-100, 1mM EDTA, 50mM pH7.5 Tris) with protease inhibitors (cOmplete protease Inhibitor Cocktail Tablets, Roche, Indianapolis, IN) and lysates were frozen at -20°C for later Western Blot analysis.

### Microarray analysis

BAT2 cells at 50% confluence were treated with BAT2 media alone or with BRSV at 0.5 MOI for 60 h at which time cells were almost confluent. This was followed by 4 h incubation with *H*. *somni* strain 2336 CCS at 20X concentration (final concentration in cell culture media) or media alone. RNA was extracted with the RNeasy minikit (Qiagen, Maryland) and gene expression was profiled using Affymetrix GeneChip bovine genome arrays, which were processed in the UC Davis School of Medicine Microarray Core Facility. The experiment was done 3 times and the data analyzed using dChip (DNA–Chip Analyszer) and DAVID (The Database for Annotation, Visualization and Integrated Discovery).

### Lipooligosaccharide (LOS) preparation and quantitation

Strain 2336 LOS (also called endotoxin) was prepared by hot phenol extraction as previously reported [23, 24]. The amount of endotoxin in CCS was determined with a Pierce LAL Chromogenic Endotoxin Quantitation Kit (Thermo Scientific, Rockford, IL) according to the manufacturer’s instructions. 1 ug/ml LOS was considered to be equal to 20,000 EU/ml as reported by others [25]. This LOS preparation was used in assays of viperin expression by BT cells to investigate the role of this *H*. *somni* toxin in stimulation of antiviral proteins as described in the Results section.

### Western blotting

*H*. *somni* CCS, live bacteria or BT/BAT2 cell lysates boiled in SDS loading buffer (Biorad, Hercules, CA) were loaded onto 10% SDS PAGE gels, run at 180V for 65 min, and transferred onto nitrocellulose membranes in a cold room at 30V overnight and 70V for an additional 1 h the next morning. Membranes were blocked with TBSTG (TBS + 0.05% Tween-20 + 0.3% gelatin) for 1 h. To detect viperin and GAPDH, membranes were incubated with primary antibodies to viperin, 1:100 (MAP-VIP, a generous gift from Dr. Peter Cresswell, Yale University) or mouse anti GAPDH, 1:4,000 (Life Technologies, Grand Island, NY) for 2 h at room temperature. Bacterial antigens were detected with convalescent phase serum (calves E5/E7, 1:1, at 1:1000) or rabbit (405) antibody to IbpA DR2 at 1:1000 [13]. After washing, membranes were incubated with alkaline phosphatase labeled goat anti mouse IgG (H+L) at 1:10,000, goat anti bovine IgG (H+L) at 1:16,000 or goat anti rabbit IgG (H+L) at 1:8,000 (Kirkegaard & Perry Laboratories, KPL, Gaithersburg, MA) for 1 h and developed in NBT/BCIP (Thermo Scientific Pierce, Rockford, IL) for 10 min.

### Bacterial adherence assay

Adherence of *H*. *somni* strains to BT cells was done because attachment to epithelial cells is sometimes a step in mucosal colonization and in biofilm formation. Continuous release of secreted factors from *H*. *somni* at the surface of the upper respiratory tract may increase the antiviral response. Since BT cells are from the upper respiratory tract and are susceptible to BRSV and *H*. *somni* infection, those cells were used in these experiments. Adherence was determined by ELISA in 96-well plates coated with 0.01% gelatin, and seeded with 1 x 10^4^ BT cells per well, as previously described, with modifications [26]. Coated wells without BT cells served as controls. Cells were grown to confluency and fixed with 0.05% L-glutaraldehyde (Sigma, St. Louis, MO) for 1 h. Previous studies showed that adherence to fixed cells was the same as to fresh cells but with less variability [26]. Any remaining L-glutaraldehyde was neutralized with 100mM glycine, and then the plates were washed and stored for up to one week at 4°C. Mid-logarithmic phase *H*. *somni* growth in BHITT, was diluted to approximately 2 x 10 ^8^ bacteria/ml by spectrophotometry and 100 ul of this bacterial suspension was added to each well of BT cells at 300 MOI. After 1.5 h incubation at 37°C, wells were washed 4 times with PBS, and fixed with 5% formalin (Sigma, St. Louis, MO) in PBS. ELISA plates were blocked with 3% gelatin in PBS with 0.02% sodium azide at 37°C overnight, incubated with convalescent phase bovine serum (calves E5+E7, 1:1) at 1:1000 dilution, followed by peroxidase labeled goat anti bovine IgG (KPL, Gaithersburg, MA) and then developed with TMB sure blue (KPL, Gaithersburg, MA). The plates were read on a dual wavelength Vmax kinetic microplate reader (Molecular Devices Corp., Menlo Park, CA) at 450/650nm. Morphology of *H*. *somni* adherence was determined by treating BT cells with live bacteria at 100 MOI in 12 well plates as above but with cells grown on coverslips in the wells. Conditions were the same as for ELISA evaluation of adherence. Coverslips were washed in the wells, fixed with 4% paraformaldehyde, stained with 0.5% crystal violet for 5 m, washed and air dried.

### BRSV proliferation in BAT2 cells

Confluent BAT2 cells in 12 well plates coated with 0.1% gelatin were treated with 1X CCS final concentration in BAT2 media in appropriate wells. Control wells were treated with BAT2 media alone. Six hours post initial CCS treatment, media or CCS treated wells were infected with BRSV at a concentration of 5.0 MOI. Additional wells with and without CCS treatment were not treated with BRSV. Cells in appropriate wells were retreated with 1X CCS at 24-h intervals for all time points longer than 24-h. Cells and supernatant were harvested at 24 h, 36 h, 48 h, and 72 h.

For PCR, BAT2 cells were lysed in triplicate by standard freezing and thawing, through the addition of 2-4ml fresh cold media and placing the plate into -80°C for 5 min, then removing the plate and placing into a 56°C water bath until thawed. The freeze/thaw supernatants were then combined with the BAT2 cell culture supernatants. Viral supernatants were then processed to extract the total RNA using the E.Z.N.A Viral RNA kit (Omega Bio-Tek), according the manufacturer’s directions. Extracted RNA was stored at -80°C until use. Viral cDNA was synthesized by using SuperScript III First Strand synthesis system (Invitrogen, CA), according to the manufacturer’s directions. The cDNA thermocycling program consisted of 10 min at 25°C, followed by 50 min at 50°C, and a termination cycle of 85°C for 5 min. Synthesized cDNA was stored at -20°C until use. The qRT-PCR was performed on a 384-well plate, in a 20 ul reaction volume. The 20 ul reaction mixture contained 10ul Sybr green qPCR master mix, 2 ul of BRSV-NP-F forward primer (GCAATGCTGCAGGACTAGGTATAAT), 2 ul BRSV-NP-R reverse primer (ACACTGTAATTGATGACCCCATTC), 2ul Nuclease free water, and 4ul of fresh cDNA product. The RT-PCR thermocycling program consisted of 48°C for 30 min, 95°C for 5 min, followed by 40 cycles of 95°C for 1 min and 55°C for 1 min. Fluorescence was measured following each cycle and displayed graphically (AB Applied Biosystems ViiA-7 detection software, version 1.1). ViiA-7 software was used to determine a cycle threshold (Ct) value, which identifies the first cycle at which the fluorescence is detected above the baseline for each sample or standard. The calculated Ct values for both GAP213DH and BRSV were analyzed against a standard curve. The standard curve was created using live virus, which was independently titered by TCID50. By using the TCID50 calculations for PFU and titers as the base line, this eliminated the dead virus from the calculation, as the TCID50 provides live viral concentrations. The cDNA prepared from the live virus was diluted 6 times using 10-fold dilutions for a final dilution of 1:1,000,000. From the serial dilution of virus cDNA the software determined the Ct value for each dilution. This Ct value was then converted into a log copy number. The log copy number for the neat viral sample was converted into a BRSV viral copy number using the calculation of live viral particles from the TCID50, this created the high point for the standard curve and this value was then diluted ten fold for 6 dilutions. By using the TCID50 value only live virus is measured. The Ct value for each sample for the housekeeping gene, GAPDH, and BRSV was then converted to a copy number using the standard curve. The copy number for BRSV was then divided by the copy number for GAPDH, to get the final value for viral shedding.

### Statistical analysis

All graphs and statistical analyses by unpaired t tests were made using GraphPad Prism, version 6. P values of <0.05 (*) and P < 0.01 (**) were considered significant. Data presented are the means and standard error of the mean (SEM).

## Results

### Induction of antiviral genes by BRSV, *H*. *somni* or dual treatment of BAT2 cells

**Fig 1:**
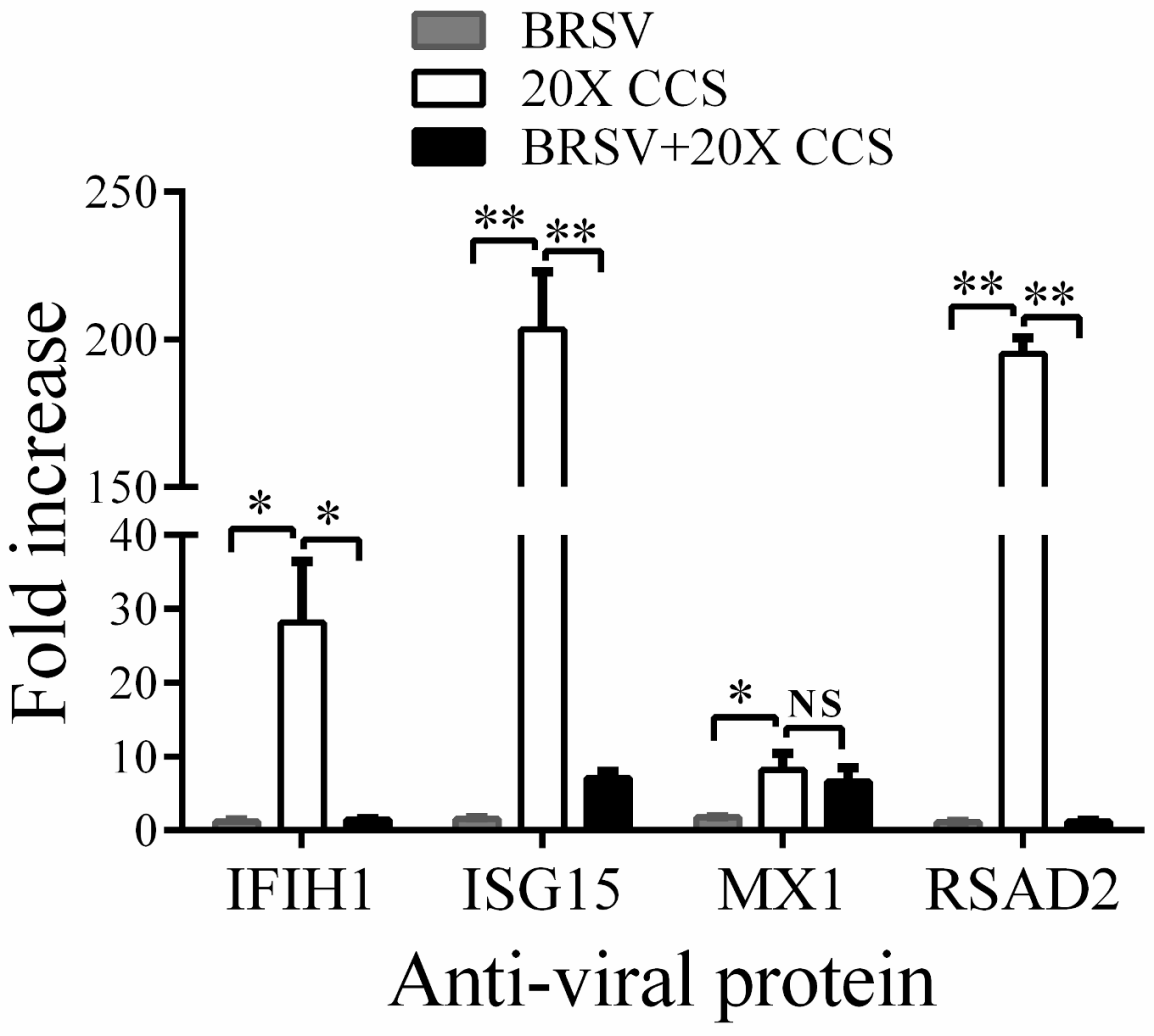
*Histophilus somni* CCS up-regulation of mRNA expressions of antiviral proteins. BAT2 cells were treated with medium alone or *H*. *somni* strain 2336 CCS (concentrated 20X) for 6h, and mRNA was purified for microarray analysis. Fold increase was calculated as the mean number of transcripts for cells treated with CCS over that of control cells treated with medium alone. Means were calculated from three independent experiments. The IFIH1 value is the mean of the 2 IFIH1 EST results in Table 1. Note that four antiviral protein genes (IFIH1, ISG15, MX1 and RSAD2 or viperin) were greatly up-regulated in CCS treated cells as compared with BRSV treated cells. Significant differences were analyzed by two tailed unpaired t test (*, P < 0.05; **, P < 0.01, NS, not significant).

We previously showed that treatment of BAT2 cells with BRSV, *H*. *somni* CCS (20X concentration) or BRSV plus CCS caused up-regulation of *mmp1* and *mmp3* genes [12], with the dual treatment causing significantly more up-regulation than BRSV or *H*. *somni* CCS alone. Therefore, we investigated other changes in gene expression by microarray analysis of BAT2 cells after exposure to BRSV, CCS or dual treatment. We found that CCS alone induces up-regulation of mRNA transcripts for antiviral genes IFIH1 (interferon induced with helicase C domain 1), ISG15, MX1 (Myxovirus resistance 1), and RSAD2, also called viperin (Table 1). Interestingly, infection with BRSV or the dual treatment of BRSV followed by CCS up-regulated antiviral gene expression much less than CCS alone. These results were of special interest because *H*. *somni* may inhibit viral infection, unlike the viral bacterial synergy which is seen when the viral infection precedes the bacterial infection. Thus, we further investigated *H*. *somni* up-regulation of antiviral genes with a view to defining the role of increased antiviral proteins in inhibition of virally induced disease. ISG15 and viperin gene expression were up-regulated the most by treatment of BAT2 cells with *H*. *somni* CCS (Fig 1). We focused on viperin for studies of up-regulation of antiviral protein expression by *H*. *somni* and for studies of mechanism of up-regulation.

**Table 1:** Microarray analysis of BAT2 cell gene expression after treatment with BRSV and/or *H*. *somni* CCS.

Gene	ETS Accession #	Entrez Gene #	# of gene transcripts after treatment with:			
			Medium	BRSV	CCS	BRSV+CCS
IFIH1*	CK940246	535490	25	26	533	38
IFIH1*	CB453859	535490	29	30	1005	43
ISG15	NM_174366.1	281871	764	1136	152115	5281
MX1	NM_173940.2	280872	103	176	751	605
RSAD2**	CB530781	506415	31	35	5971	37

*Two different IFIH1 ESTs from the same gene.

**RSAD2 is also called viperin. Data presented equal means calculated from three independent experiments.

### Up-regulation of viperin protein expression in cells treated with *H*. *somni* or its secreted products

**Fig 2:**
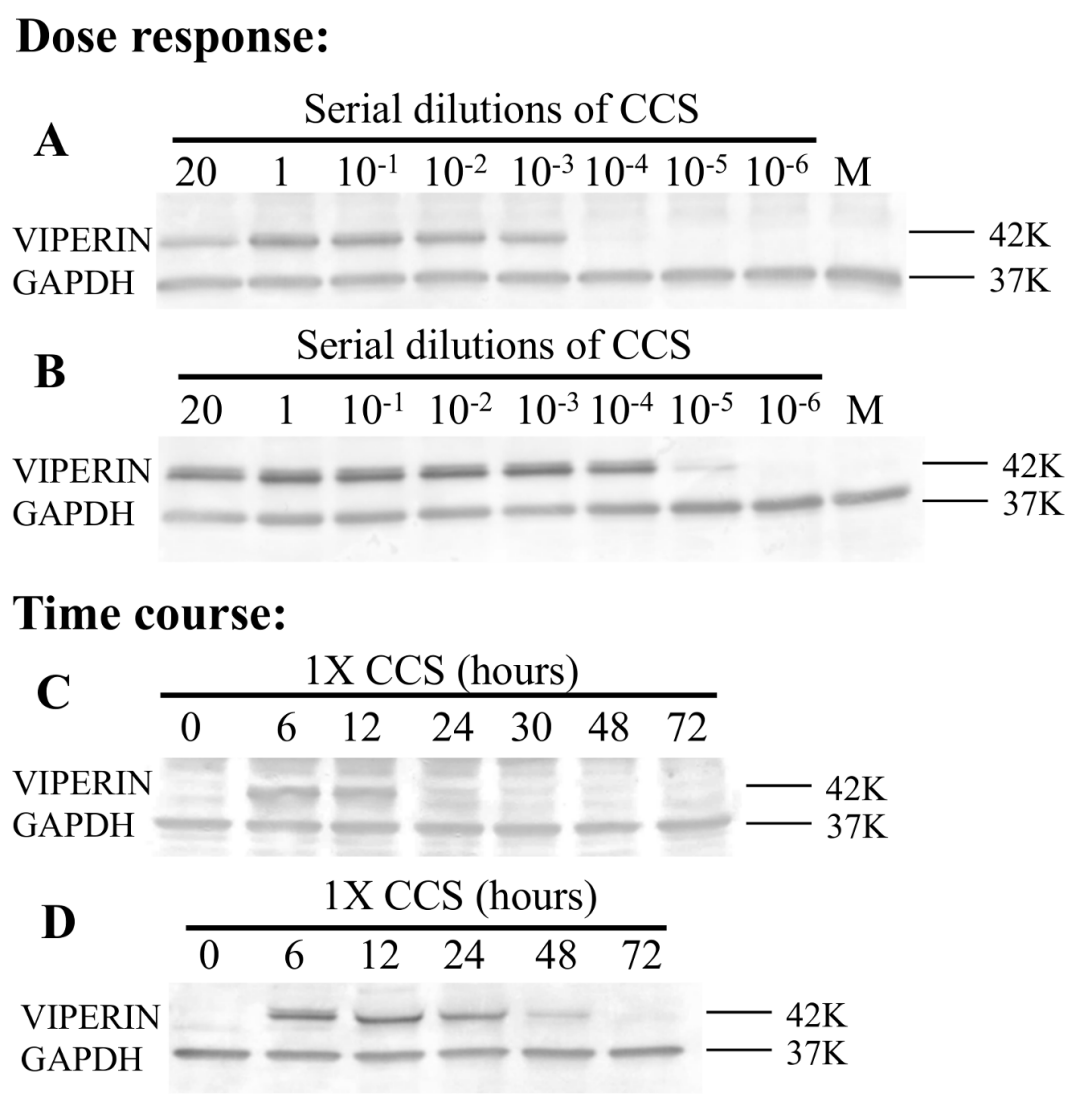
Dose response and time course of *H*. *somni* 2336 induction of viperin protein expression. For the dose response: BT (A) or BAT2 (B) cells were incubated with dilutions of *H*. *somni* 2336 CCS (concentrated 20X) or with BHI medium (M). Cells were lysed at indicated time points. For the time course: BT (C) or BAT 2 (D) cells were treated with *H*. *somni* 2336 1X CCS or medium (M) alone and cell lysates were collected after 6 h. In each case, cell lysates were separated by SDS PAGE and Western Blots reacted with antibody to viperin and to GAPDH. Controls with dilutions of concentrated BHI did not induce viperin expression (data not shown). Molecular weight markers are on the right.

The kinetics of viperin induction by CCS was determined for both bovine upper respiratory and lower respiratory cells (bovine turbinate and alveolar epithelial cells) because *H*. *somni* can be carried in the upper respiratory tract but is found primarily in the alveolus during experimental pneumonia [4] and because BRSV infects both cell types. Although functional studies and microarray studies with BAT2 cells were done by stimulating cells with CCS concentrated 20 times [12, 13], dose response studies showed that this CCS could be diluted at least 1000 times before less viperin protein expression was detected in BT cells or BAT2 cells by Western blotting (Fig 2A and 2B). Therefore, subsequent studies used 1X CCS (equivalent to undiluted culture supernatant, which should be physiologic). Preliminary studies using the trypan blue exclusion method, showed that this concentration did not kill or inhibit proliferation of BT cells. The time course of viperin protein expression was then studied by stimulating cells with 1x concentrated CCS (equivalent to undiluted culture supernatant). Western blots of cell lysates showed that viperin protein expression was detectable in BT cells for at least 12 h (Fig 2C) and in BAT2 cells for at least 48 h after addition of CCS (Fig 2D). Thus, *H*. *somni* CCS up-regulates viperin protein expression in a dose dependent and time dependent manner in both BT and BAT2 cells.

### Effect of antiviral proteins on BRSV replication

**Fig 3:**
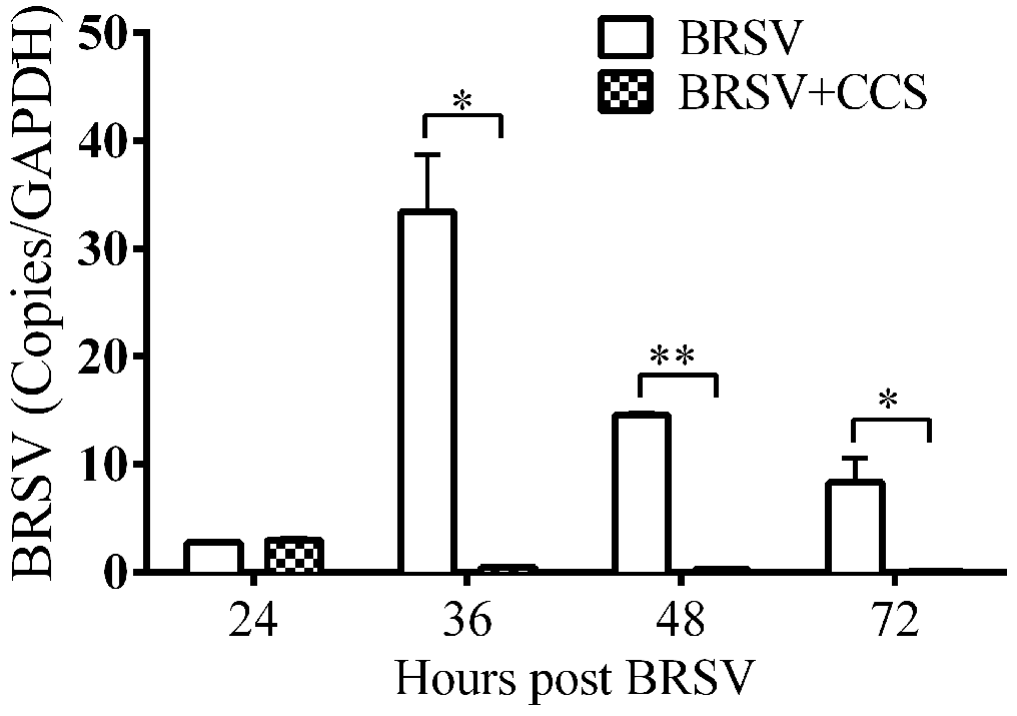
Effect of *H*. *somni* culture supernatant on BRSV replication in BAT2 cells. Cells were treated with 1X CCS or BAT2 control medium at the time of BRSV infection (0 time) and at 24 and 48 hours post BRSV infection based on the time course of viperin expression shown in Fig 2D. BRSV proliferation determined by qRT-PCR was depressed after treatment of cells with CCS. Significant differences were analyzed by one-tailed unpaired t test (*, P < 0.05; **, P < 0.01).

Up-regulation of antiviral proteins in respiratory epithelial cells should result in decreased viral load in cells treated with *H*. *somni* or its culture supernatant, if the protein is functional. We tested this hypothesis in by treating BAT2 cells with *H*. *somni* CCS before infecting with BRSV since viperin protein expression was increased for longest in that cell type. BRSV was detected by qRT PCR of BAT2 control cells by 36 hours but only at very low levels of BRSV were detected in cells treated with *H*. *somni* culture supernatant throughout the 72 h study (Fig 3). So pretreatment of BAT2 cells with *H*. *somni* secreted products greatly depressed BRSV replication.

### Virulence factors in *H*. *somni* CCS

**Fig 4:**
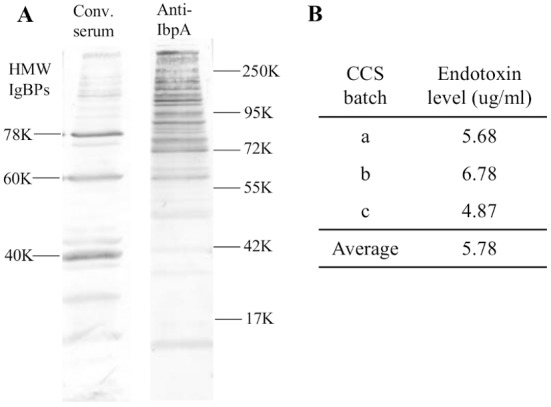
Virulence factors of *H*. *somni* released into the culture supernatant. (A) Western blotting of strain 2336 CCS against convalescent phase serum (from calves E5 and E7) or rabbit antibodies to IbpA DR2 showing many high molecular weight IgBPs reacting slightly with the convalescent phase serum but strongly with anti-IbpA as well as immunodominant 78K, 60 K and 40 K antigens. Antigens are labeled on the left and molecular weight markers on the right. (B) Endotoxin levels in 3 batches of strain 2336 CCS (at 1 x concentration) as determined by LAL assay.

**Fig 5:**
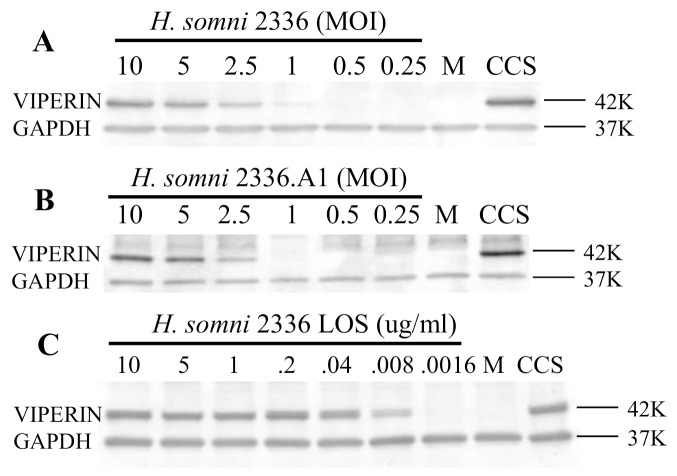
*Histophilus somni* toxins involved in stimulating viperin expression in BT cells. (A) Pathogenic strain 2336 and (B) the IbpA deleted strain (2336 A1) live bacteria induce viperin expression equally as shown by Western Blotting of cell lysates after 6 hrs of treatment. Therefore, IbpA is not necessary to for up-regulation of viperin. (C) Purified *H*. *somni* strain 2336 lipooligosaccharide (LOS) induces viperin expression even at very low doses, showing that LOS is sufficient to up-regulate viperin expression. All compared with treatment by medium (M) alone or strain 2336 CCS as negative and positive controls, respectively. Molecular weight markers are on the right.

Pathogenic *H*. *somni* strain 2336 culture supernatant is potent in stimulating antiviral proteins expression. Since *H*. *somni* releases two known toxins (LOS or endotoxin and the IbpA cytotoxin), we hypothesized that one of these factors in CCS may be stimulating viperin expression. BT cells were used in these experiments since inhibition of BRSV or other respiratory virus in the upper respiratory tract could result in fewer virus particles reaching the lung to produce pneumonia. Western blotting against convalescent phase serum showed that CCS contained the high molecular weight “ladder” of immunoglobulin binding proteins (IgBPs) as is characteristic of the IbpA cytotoxin as well as immunodominant 78, 60 and 40 K antigens which have not been shown to be cytotoxic (Fig 4A). Antibodies to IbpA DR2 revealed many IbpA bands from above 250 K to below 60 K, perhaps at least partly due to the multiple start codons in the IbpA gene as demonstrated earlier [27, 28]. The amount of endotoxin or LOS (the second cytotoxin) was determined by LAL assay in 3 different CCS preparations (which had been used for cytotoxicity assays in the past). Average levels of LOS in CCS were shown to be about 5.8 ug/ml (Fig 4B). To determine whether one of the two toxins (IbpA cytotoxin or LOS endotoxin) were involved in viperin induction, we compared BT cells challenged with live *H*. *somni* 2336 and the homologous strain with *ibpA* gene deleted (2336.A1) [22] at 10 MOI.Both the wild type (2336) and the knockout (2336.A1) induced viperin protein expression in a similar dose dependent manner, showing that the IbpA gene and protein were not necessary for *H*. *somni* up-regulation of viperin expression (Fig 5A and 5B). To determine whether *H*. *somni* LOS stimulated viperin, BT cells were treated with LOS extracted from *H*. *somni* strain 2336. Treatment of BT cells with serial dilutions of purified LOS from strain 2336 showed that as little as 8 ng/ml LOS induced a detectable amount of viperin as determined by Western blotting (Fig 5C). This is comparable to the amount of endotoxin in the highest dilution of CCS (10^−3^) which induced viperin expression (Fig 2), or 5.8 ng/ml as calculated from the LAL assay (Fig 4B). Whether other factors in CCS, such as *H*. *somni* OMPs, might contribute to up-regulation of viperin expression was not determined.

**Fig 6:**
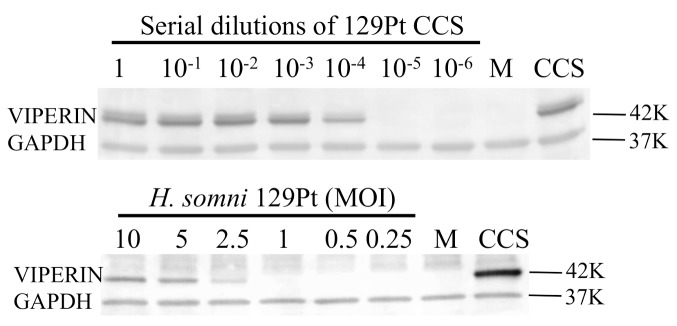
Viperin expression in BT cells after treatments with the IbpA negative asymptomatic carrier strain of *H*. *somni* strain129Pt. Both CCS and live strain 129Pt induced viperin expression as determined by Western blotting. Media (M) and strain 2336 CCS were included as negative and positive controls, respectively. Molecular weight markers are on the right. Viperin induction by strain 129Pt CCS or live strain 129Pt was comparable to that of strain 2336 in Figs 2 and 5 respectively.

**Fig 7:**
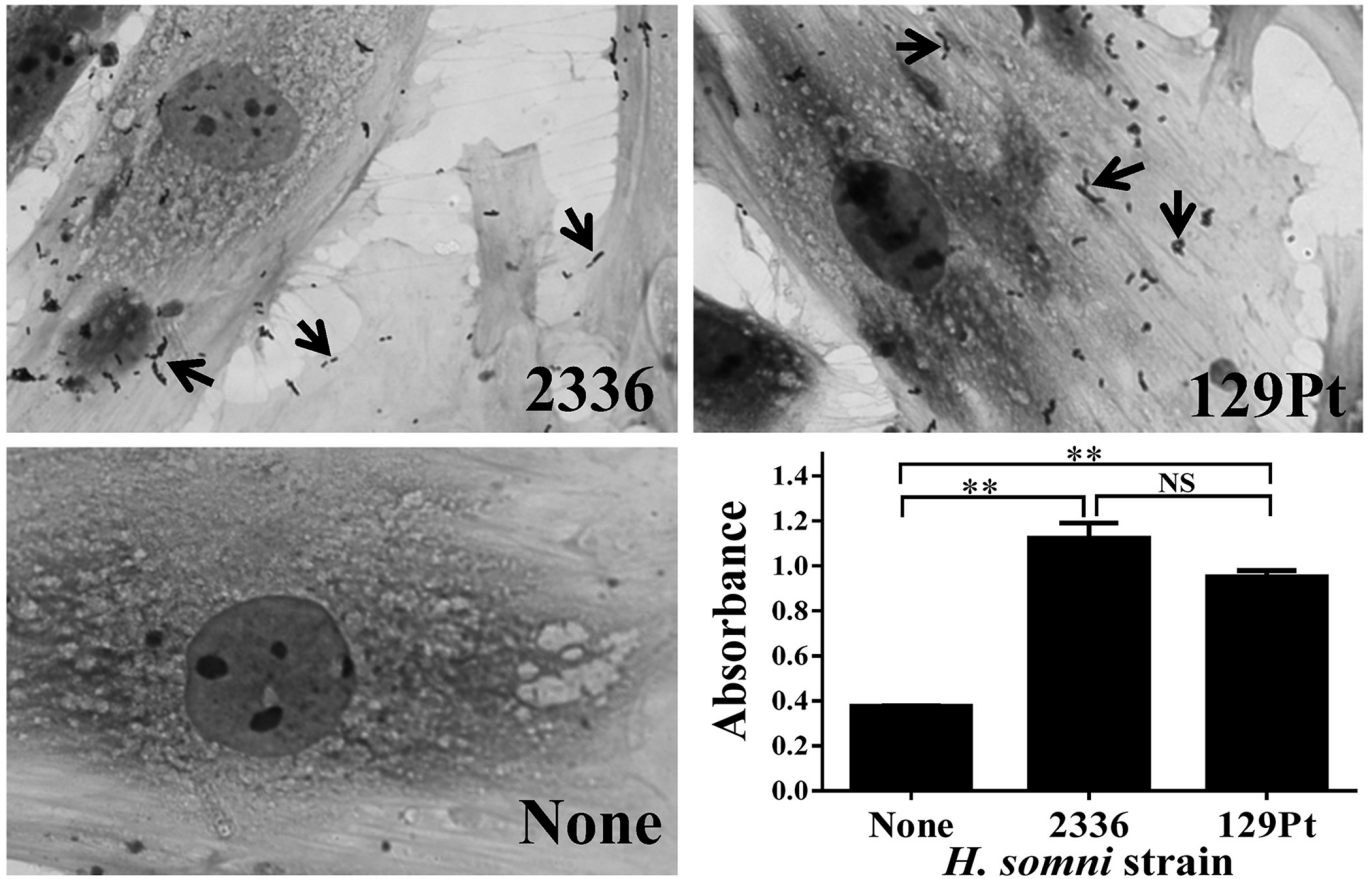
Adherence of *H*. *somni* strain 2336 and 129Pt to BT cells. Live bacteria were co-incubated with BT cells at 300 MOI in 96-well plates for 2 h, free bacteria were washed off and adherence determined by ELISA with *H*. *somni* specific antibody. The morphology of adherence is shown in Giemsa stained BT cells on coverslips treated with 100 MOI *H*. *somni* 2336, 129Pt or no bacteria. Arrows indicate adhered bacteria. Although there was variation from field to field, adherence of both strains was similar. Significant differences were analyzed by two-tailed unpaired t test (**, P < 0.01, NS, not significant).

The ability of a naturally occurring *ibpA* negative strain 129Pt [17] to stimulate viperin expression in bovine upper respiratory epithelial cells was then determined since this strain was isolated from the epithelium of an asymptomatic carrier bull [29]. An *ibpA* negative strain was chosen because it lacks a critical virulence factor, so would not cause disease. Live *H*. *somni* 129Pt at a low MOI (2.5) induced viperin expression, and CCS from strain 129Pt at a 1:10,000 dilution induced as strong a viperin protein expression in BT cells as strain 2336 (Fig 6A and 6B compared with Figs 2A and 4A). These were similar results to those with the virulent strain 2336. Since colonization of the bovine upper respiratory tract with viperin inducing, non-virulent *H*. *somni* may induce antiviral protein expression and attachment is one step in colonization by biofilm formation, we examined attachment of strains 129Pt and 2336 to BT cells *in vitro*. Both microscopy (Fig 7A, 7B and 7C) and ELISA based assays (Fig 7D) showed that *H*. *somni* strain 129Pt adheres to BT cells approximately as well as the pathogenic strain 2336.

## Discussion

This study shows that *H*. *somni* live bacteria and culture supernatant stimulate an antiviral response in the bovine upper and lower respiratory tract epithelial cells. This has implications for host resistance since bacterial/viral interactions are critical to the etiology of this polymicrobial disease, and respiratory epithelial cells are a first line of defense. All or some of the four up-regulated antiviral proteins in this study (IFIH1, ISG15, MX1 and RSAD/viperin) also were shown to be up-regulated in epithelial cells by cigarette smoke, or several viruses [30–34]. In our studies, *H*. *somni* up-regulated the antiviral response strongly, but BRSV infection alone up-regulated it only a little, if any. Surprisingly, BRSV, followed by *H*. *somni* CCS, down-regulated these antiviral genes as compared with CCS alone. This suggests that antiviral responses may be up-regulated if the bacterial infection occurs first but down regulated if the BRSV infection occurs first. Although many viruses and some bacterial products have been shown to induce an antiviral response, there has been little investigation of the effect of bacterial stimulation of antiviral proteins on viral infection.

Induction of bacterial inhibition by bacterial respiratory pathogens has been demonstrated previously. We showed that some isolates of bovine nasal flora inhibit *in vitro* growth of bovine bacterial respiratory pathogens, others enhance pathogen growth while still others have no effect [35]. Other investigators found that human bacterial pathogens, such as *Streptococcus pneumoniae and Haemophilus influenzae*, compete with each other *in vitro and in vivo* [2]. Viral predisposition to bacterial infection has been reported by our team and by others [2, 11, 12]. However, bacterial influence on viral infection is less well investigated. Bosch et al [1] cite several studies that suggest a preceding bacterial infection may increase susceptibility to a subsequent viral infection. Our studies indicate that *H*. *somni* infection of respiratory epithelial cells may have the opposite effect on BRSV infection. With a qRT-PCR assay based on the dose response and time course of viperin protein expression, we showed that the antiviral response induced by secreted factors of *H*. *somni* was associated with decreased production of BRSV by BAT2 cells. Many studies have shown that antiviral responses initiated by many DNA and RNA viruses do inhibit viral production [36–39]. For example, replication of human RSV virus in respiratory tract cells is inhibited by viperin *in vitro and in vivo* [34]. Large numbers of interferon stimulated genes were screened recently for antiviral activity against a panel of 14 viruses [40]. Infectivity of RSV was inhibited by several ISGs. The roles of ISG15 and RSAD2 *in vivo* and *in vitro* have been reviewed [41]. Most of the *in vivo* and *in vitro* ISG studies involved single genes investigated by ectopic expression or gene deletion. Although we did not do combinatorial studies of gene networks, our studies did look at several antiviral proteins expressed together in response to bacterial stimulation. The fact that this increased expression of several antiviral genes occurred when target respiratory epithelial cells from the natural host were treated with shed factors from bacteria involved in the disease complex suggests that the results may be relevant to disease prevention. Others have shown that infection with *Salmonella typhimurium*, *Listeria monocytogenes*, *Lactobacillus acidophilus* or *Streptococcus agalactiae* resulted in up-regulation of viperin expression by macrophages or dendritic cells but the effect on viral replication was not tested and epithelial cells responses were not examined [42–44]. Little information is available on induction of antiviral proteins by bacteria with subsequent inhibition of viral replication *in vitro* or *in vivo*. Therefore, we investigated the mechanisms of *H*. *somni* induction of antiviral responses, showing that *H*. *somni* LOS but not IbpA increased viperin protein expression. Both are toxic for bovine respiratory tract epithelial and endothelial cells [13, 15, 45]. We expected that IbpA may be involved in the antiviral response, since *Bordetella pertussis* FHA stimulates human peripheral blood mononuclear cells to up-regulate the ISG15 antiviral protein gene [46] and *H*. *somni* IbpA and FHA are quite homologous in the N terminal region. This was not the case. The C terminal portion of *H*. *somni* IbpA, with the two fic toxic domains, has no homology to FHA and was also not associated with the antiviral response. On the other hand, very low amounts of *H*. *somni* endotoxin (LOS) stimulated strong viperin responses in a dose dependent manner. This is consistent with other studies showing that endotoxin or lipopolysaccharide is a strong inducer of antiviral proteins, including IFIH1, ISG15, MX1 and viperin [47, 48] but this has not been extended to studies of *in vitro* or *in vivo* inhibition of viral replication.

Since we showed that *H*. *somni* strain, 2336.A1, lacking the critical IbpA fic cytotoxic virulence factor, could stimulate a strong antiviral response, we determined whether a naturally occurring *ibpA* negative isolate from an asymptomatic carrier would have a similar effect. Future attempts to introduce a naturally occurring *ibpA* negative strain may be more acceptable than a genetically modified strain and an *ibpA* negative strain would lack the ability to cause invasive disease. We previously showed that four naturally occurring asymptomatic strains which lack the *ibpA* gene [16], were serum sensitive, did not cause cytotoxicity and were likely non-pathogenic members of the mucosal microbiome. We chose to study strain 129Pt, from among these four, because it has been well characterized and its genome fully sequenced [17]. It does not cause retraction of BAT2 cells, unlike IbpA positive strains [13, 15]. Both CCS and live bacteria of strain 129Pt stimulated strong viperin protein production in BT cells. We determined the ability of strain 129Pt to adhere to BT cells, as an example of bovine upper respiratory tract cells because adherence is the first step in biofilm formation. Attachment and biofilm formation in the upper respiratory tract with *ibpA* negative strains should result in release of LOS over time *in vivo*, and up-regulation of antiviral proteins to inhibit BRSV and perhaps other respiratory viral infections. This may occur naturally in cattle carrying *H*. *somni* in the upper respiratory tract and may partially explain individual animal differences in susceptibility to respiratory infection. Although biofilm formation by *H*. *somni* strain 129Pt in the upper respiratory tract of cattle has not been tested experimentally, the *in vitro* results provide hope that it may be possible to introduce a bovine asymptomatic carrier isolate of *H*. *somni* (like strain 129Pt) to the upper respiratory microbiome of calves by intranasal inoculation to stimulate antiviral responses and decrease susceptibility to respiratory disease. Lactobacilli are often used as probiotics in the gut, and *Lactobacillus acidophilus* induces strong antiviral protein gene up-regulation [43]. Maybe a similar approach could be used prophylactically in the upper respiratory tract.
